# Safety and Dose-Dependent Effects of *Echinacea* for the Treatment of Acute Cold Episodes in Children: A Multicenter, Randomized, Open-Label Clinical Trial

**DOI:** 10.3390/children7120292

**Published:** 2020-12-15

**Authors:** Ramon Weishaupt, Arnold Bächler, Simon Feldhaus, Günter Lang, Peter Klein, Roland Schoop

**Affiliations:** 1 A. Vogel AG, Grünaustrasse 4, 9325 Roggwil, Switzerland; r.weishaupt@avogel.ch; 2 Pediatric Practice, Notkerstrasse 14, 9000 St. Gallen, Switzerland; arnold.baechler@hin.ch; 3 Paramed Ambulatory, Paramed AG, Haldenstrasse 1, 6340 Baar, Switzerland; s.feldhaus@paramed.ch; 4 General Practice, Burgstrasse 112, 4125 Riehen, Switzerland; guenter.lang@gmx.ch; 5 d.s.h. Statistical Services GmbH, Turmbergweg 5, 85296 Rohrbach, Germany; Peter.Klein@dsh-statistik.de

**Keywords:** *Echinacea*, Echinaforce, fresh plant, phytotherapy, common cold, respiratory tract infects, dose response, antiviral/immunomodulatory activity, children, recurrences

## Abstract

**Background:** Due to the frequency and severity of cold symptoms in children, and the risk of associated complications, effective treatments are urgently needed. Here we evaluated the safety profile and treatment benefits of *Echinacea* in children with acute cold and flu symptoms. **Methods:** A total of 79 children (4–12 years) were randomized to a treatment regimen of three or five times daily Echinaforce Junior tablets (total of 1200 or 2000 mg *Echinacea* extract, EFJ) for the prospective treatment of upcoming cold and flu episodes at first signs. Parents recorded respiratory symptoms daily during episodes in their child and physicians and parents subjectively rated tolerability. **Results:** EFJ was used to treat 130 cold episodes in 68 children and was very well tolerated by more than 96% positive physician’s ratings. EFJ-treated cold episodes lasted 7.5 days on average, with nine out of 10 episodes being fully resolved after 10 days. Five EFJ tablets daily reduced the average episode duration by up to 1.7 days (*p* < 0.02) in comparison to three EFJ tablets daily regimen. Effective symptom resolution finally contributed to a low antibiotic prescription rate in this study of 4.6%. **Conclusions:** EFJ tablets present a valuable option for the treatment of acute cold episodes in children showing a wide safety margin and increased therapeutic benefits at five tablets daily.

## 1. Introduction

Acute respiratory tract infections (RTIs), like the common cold, constitute the most frequent illnesses in Western civilization [1,2]. They continue to represent a tremendous societal burden in terms of morbidity and economic losses [3]. Although colds are mostly benign and self-limiting, they result in over 20 million consultations to primary care, and over 40 million lost school and work days per year in the US alone [4,5].

Children are particularly susceptible to colds, experiencing 6–12 episodes per year compared to 2–4 annual episodes among adults [3,6]. Children also tend to develop more severe symptoms and exhibit a higher complication rate [7]. Approximately 70% of school absences and 85% of asthma exacerbations are related to common colds [4]. In children, symptoms last substantially longer than in adults, 15 days on average, and certain symptoms (e.g., cough) can persist for up to 25 days [8,9].

Conventional therapeutics largely rely on antipyretics, decongestants, and cough suppressants, which are often, administered “off-label” as over-the-counter remedies. Although such medications are generally expected to rapidly improve the comfort of an ill child, no effect on illness duration has been proven [10,11]. Moreover, serious safety concerns have been raised about the use of such medications in young children [12,13,14]. Antimicrobials have been proven ineffective against colds, and carry risks of resistance development and side effects, but are still frequently prescribed [15,16].

The purple coneflower (*Echinacea purpurea*), a medical plant originating in North America, and preparations thereof have a long history for the acute treatment and prevention of colds and flus [17]. Standardized hydro-ethanolic extracts (tinctures) prepared from freshly harvested *Echinacea purpurea* (95% herb and 5% roots) exhibit strong inactivation activity against viruses involved in RTIs, without the risk of resistance development [18,19]. Anti-inflammatory and immunomodulatory activities may contribute to the effectivity profile of *Echinacea* [20,21].

In the present study, we tested the safety and treatment effects of Echinaforce^®^ Junior (EFJ) tablets, an *Echinacea* extract-containing remedy that has been formulated to be suitable for use by patients below 12 years of age. The goal of this clinical study was to assess the safety and to systematically investigate whether an increased daily EFJ dose could reduce the duration and severity of cold episodes in children under real-life conditions. Evidence of effects was obtained by comparing two dosage groups (3 × 1 EFJ tablets/day vs. 5 × 1 EFJ tablets/day) to elaborate any dose response effects. No child was exposed to the risk of non-treatment and all participants were optimally supplied as stipulated by Gómez-Díaz et al. (2011) [22].

## 2. Materials and Methods

### 2.1. Participants

Principally healthy children of 4–12 years of age were recruited and included upon receipt of informed consent signed by their parents (and optionally by the child). The following exclusion criteria applied: Age of ≥13 years or <4 years; presence of or predisposition to complicated respiratory infections at inclusion visit; participation in clinical trials within the last 30 days; intake of antimicrobial, antiviral, or immune-suppressive medication; intake of salicylic substances or broncho-vaxom; surgical interventions within the last 3 months; known diabetes mellitus, allergic rhinitis, atopy, asthma, cystic fibrosis, bronchopulmonary dysplasia, immune system diseases, degenerative disorders (e.g., autoimmune diseases, such as AIDS or leucosis), metabolic or resorption disorders, liver or kidney disorders, other serious health conditions; known allergy to composite plants (chamomile or dandelion) or any compound of the test medication. Trial registration: Clinicaltrials.gov: NCT02198391.

### 2.2. Formulation

One Echinaforce Junior^®^ (EFJ) tablet contains 400 mg of hydro-ethanolic extract (65% *v*/*v*) of freshly harvested *Echinacea purpurea*. Tinctures from the herb (drug extraction ratio 1:12) and from the roots (drug extraction ratio 1:11) are combined at a ratio of 95% to 5%, supplemented with excipients, and manufactured into EFJ tablets under GMP conditions. EFJ tablets were packaged into dark brown glass bottles containing either 40 or 60 tablets per bottle. One patient box contained three glass bottles, each sufficient for 10 days of treatment, together with a thermometer (Medisana TM750, Neuss, DE, Germany). EFJ tablets were batch released for low-dose (No. 040641A) and high-dose (No. 040641B) groups, and delivered to the study centers in multiples of randomization packages, containing four patient boxes each, all by A. Vogel AG (Roggwil, CH, Switzerland).

### 2.3. Study Design and Patient Recruitment

This study was designed as a randomized, parallel grouped, open-label multicenter clinical trial according to ICH-GCP guidelines and reported adhering to the CONSORT guidelines.

The study was advertised in local newspapers. In order to increase the incentive of potentially interested parents/children to participate in this study, a prospective comparison of two different dosages of the study treatment was chosen instead of a direct comparison with no-treatment or placebo. Eligible children/parents were recruited by 10 pediatricians at eight participating pediatric practices across Switzerland. After informed consent form (ICF) was signed by at least the parents, children were randomly allocated to either the EFJ “low-dose” group (3 × 1 tablets daily) or “high-dose” group (5 × 1 tablets daily). Eligible children underwent a physical examination, and concomitant medication/disease assessment within the frame of a general anamnesis. Included children/parents received an access password and instructions on how to use individual online e-diaries (provider: INPADS GmbH, Bad Dürkheim, Germany) to record daily symptoms. Included children/parents obtained the investigational medicinal product (IMP), and children with acute respiratory symptoms at inclusion started treatment immediately. In all other cases, parents were asked to contact the permanently accessible study coordinator (medical practitioner) for confirmation of cold diagnosis in their child.

### 2.4. Assessments

Each day during active cold episodes, the symptoms of running nose, stuffed nose, sneezing, cough, shivering, malaise, headache/limb pain, and sore throat were to be rated as “severe” (3), “moderate” (2), “mild” (1), “absent” (0), or “not assessable” (X). Additional variables-including sleep quality and additional care effort were rated following the same criteria and the body temperature was recorded in °C by means of an electric thermometer. For analysis, ratings of “not assessable” (X) were replaced with “mild” (1). A daily symptom score (SSc) was recorded for single symptoms, and a total daily symptom score (TSSc) was calculated by combining all symptom scores. Concomitant treatments, concomitant conditions, and incidence of adverse events (AEs) were retrieved via the study coordinator during routine contacts, from the investigator during interim visits, from close-out visits, or via assessment of e-diaries. All findings and AEs were coded according to the MedDRA (version 17.0GE). Concomitant medication/therapies were coded according to the WHO ATC (2013).

At the final visit 2, returned study medication was counted to assess compliance, and the children underwent a physical investigation and final assessment of the safety and effectiveness variables. After each treatment cycle and at final visit 2, global subjective tolerability was rated by physicians and parents/children, and subjective effectiveness was rated by parents/children. Both could be rated as “very good” (1), “good” (2), “moderate” (3), or “poor” (4). Acceptance was determined by asking parents/children at visit 2 whether they would like to use the medication again. The applied methods were adapted from Taylor et al., who described a valid method for evaluating viral respiratory infections in children below 12 years old [23].

### 2.5. Randomization and Sample Size Calculations

A randomization list with a block size of 4 was prepared by d.s.h. statistical services (Rohrbach, Germany) using the validated Rancode Professional 3.6 software (IDV, Gauting, Germany). The randomization ratio was 1:1 with respect to low-dose and high-dose groups. At the study sites, the medication was distributed to each participant in accordance with this randomization list.

For effectiveness analysis, sample size was calculated for an estimated difference of 1 score point in the TSSc between the two EFJ dosages, and a standard deviation of 1.5–2.5 points. A sample size of 100 children per dosage group was estimated to achieve a statistical power of 80–99% using a two-sided t-test with α = 0.05 (nQuery Advisor Version 7.0 (Statistical Solutions Ltd., Cork, Ireland)).

### 2.6. Data Evaluation

The investigator assessed tolerability on final visit 2 as the primary endpoint. Kaplan–Meier analysis at episode level was performed to compare the episode duration and using Wilcoxon rank-sum test (time until patients were free of symptoms) between dosing groups. The end of an episode was defined as the day from which on all symptoms were rated “absent” (0) or “mild” (1) without re-increase over at least 4 days according to Kurugöl et al. [24]. When episodes had not ended until day 10, duration was censored at day 10 and the patient was considered a non-responder. The numbers of cold episodes were compared between groups. For each treatment group, the individual and total symptom severity scores (SSc and TSSc) were presented as daily averages. Any missing value was replaced by the last observation carried forward (LOCF procedure). The cumulative number of sick days were calculated during the study period for both treatment groups overall, and per included child on average.

Baseline characteristics were compared between the two dosage groups using Mantel–Haenszel (MH) tests for ordinal variables, Wilcoxon and Fisher’s exact (FE) tests for dichotomous or categorical variables, and t-tests for continuous variables. Safety variables were analyzed in the safety collective (SAF). Analyses of effectiveness parameters were conducted in the intention-to-treat (ITT) collective and the as-treated collective (AT). The AT collective was formed from the ITT collective to adjust for the effective daily IMP intake deduced from compliance. Consequently, for AT analysis, 5 patients with significantly higher IMP intake (>160 % compliance) were transferred from the low dose to the high dose group. All statistical tests were two-sided at a 5% level of significance, and were interpreted in a descriptive-exploratory way. All statistical analyses were performed using the SAS^®^ system (Version 9.3) and Testimate 6.5 (IDVGauting/München, DE, Germany).

## 3. Results

**Figure 1:**
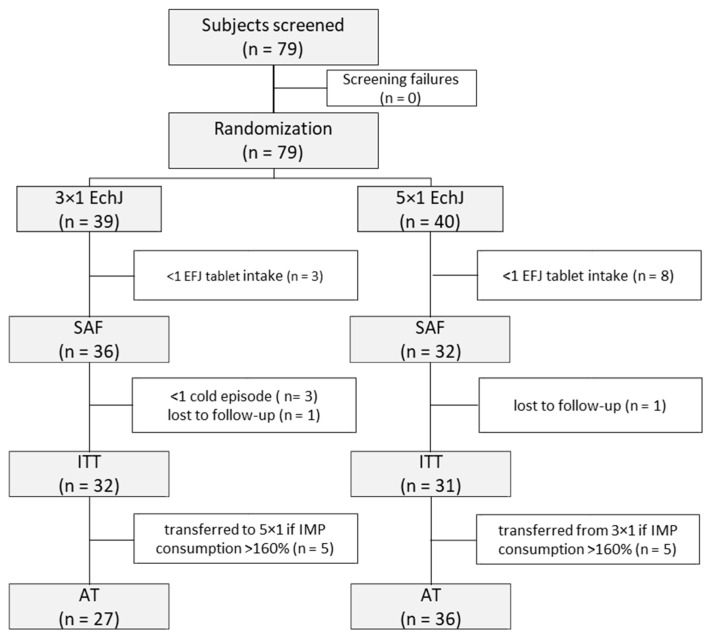
Patient disposition chart. SAF—safety collective; ITT—intention-to-treat collective; AT—as-treated collective.

This study was conducted from October 2014 to August 2015. A total of 79 children were recruited, enrolled, and randomized (Figure 1). Two out of 79 randomized subjects (2.5%) dropped out: The reason was that the children did not appear at the final visit (lost to follow-up). Overall, 130 cold episodes were reported for 68 children during 5.3 months of observation in this study. A total of 68 episodes were recorded for children in the 3 × 1 tablets group and 62 episodes for children in the 5 × 1 tablets/day group (SAF).

Table 1 lists the demographic and clinical characteristics of the included children. The overall mean age of children in the safety collective was 6.7 ± 2.4 years, whereas 34 patients (69.8%) were ≤7 years old. The gender distribution was equal in both dosage groups. Despite differences in age and body variables, children of both study groups were similar with regards to the number of experienced past cold episodes per season and hence, their susceptibility to colds as per medical history.

### 3.1. Clinical Safety Outcomes

**Figure 2:**
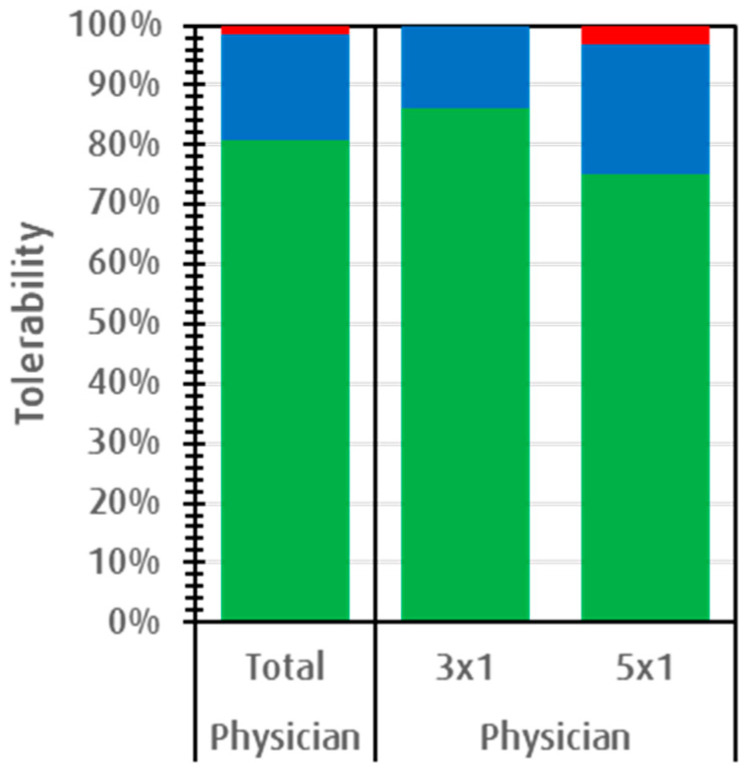
The primary endpoint of “tolerability assessed by physician” in the total safety collective and in each dosage group. Green indicates “very good”, blue “good”, and red “poor”.

Figure 2 shows that 98.5% of physicians rated the tolerability of EFJ treated episodes as “good” or “very good” in the safety collective (SAF), 100% in the low-dose group, and 96.95% in the high-dose group (MH test, *p* = 0.231). Similar positive ratings were obtained in the ITT collective.

Parents/children confirmed the physicians’ overall positive tolerability assessment, with >99% giving ratings of “good” or “very good” in both dosage groups (MH test, *p* = 0.497). Of 68 safety ratings, only one (1.5%) in the higher dose group was attributed as “poor”.

### 3.2. Clinical Effects

#### 3.2.1. Duration of Episodes

**Figure 3:**
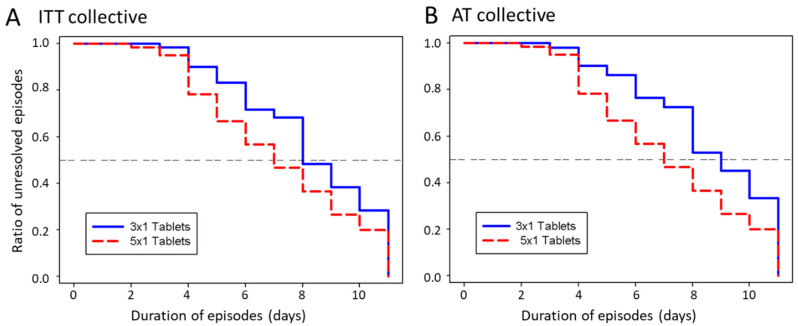
Kaplan–Meier estimate curves of the ratio of unresolved episodes per Echinaforce Junior Tablets (EFJ)-treatment day in the “intention-to-treat” (ITT) collective (**A**), and in the “as-treated” collective (**B**). The curves of high dosage group is indicated in red dashed lines and the curves of the low dosage group indicated in blue solid lines. The high-dose group in the “as-treated” collective includes five patients who were originally allocated to the low-dose group but who showed >160% EFJ consumption.

Among EFJ-treated children, cold episodes lasted an average of 7.5 ± 3.5 days (Figure 3). Compared to the lower 3 × 1 tablet dosage, 5 × 1 tablets reduced the average duration of cold episodes by 1.2 days faster (Table 2; Wilcoxon rank-sum test, *p* = 0.046). Correction for the actual study medication intake (AT collective) further accentuated the treatment benefits for the higher dosage from 1.2 days to 1.7 days (Wilcoxon rank-sum test, *p* = 0.02).

After 10 days EFJ treatment, 102 of the total 120 cold episodes (85%) in the ITT collective resolved completely overall. Twelve episodes (19.7%) in the low-dose group and six episodes (10.2%) in the high-dose group continued with at least moderate symptoms beyond 10 days. Again, the dose response was more pronounced in the AT collective, where 12 episodes (23.5%) in the low-dose group remained unresolved after treatment compared to six episodes (8.7%) in the high-dose group (Wilcoxon test, *p* = 0.005).

#### 3.2.2. Cumulative Number of Sick Days

Throughout the time of observation, children experienced a total of 494 cumulative sick days in the low dose group, and 410 days in the high dose group (ITT, ratio: 0.83; Chi-square test, *p* = 0.022). The average cumulative number of sick days per patient during the observation period were slightly reduced by 1.4 days in the ITT collective (Wilcoxon test, *p* = 0.362) and up to 2.9 days per patient in the AT collective, with five tablets daily in comparison to three tablets daily (*p* = 0.048). The dose response effect was again more pronounced in the AT collective regarding all assessed variables (Table 2).

EFJ-treated children in this study experienced an average of 1.9 episodes, which is below the reported average of 3.1 episodes during past seasons (Table 1) without EFJ (Wilcoxon rank-sum test, *p* < 0.001).

#### 3.2.3. Symptom Development

**Figure 4:**
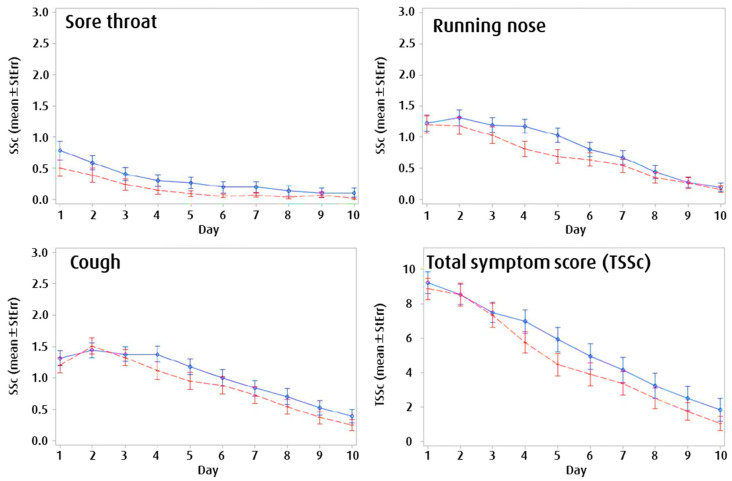
Single symptom scores (SScs) for sore throat, cough, and running nose (ITT: Intention to treat collective). Total daily symptom score (TSSc) was calculated by aggregating the severity scores of all measured cold symptoms. The course of symptoms is indicated with solid blue lines for children in the low-dose group (3 × 1 tablets daily), and with red dashed lines for children in the high-dose group (5 × 1 tablets daily).

Figure 4 shows that sore throat, running nose, and cough were more potently reduced in children with the higher EFJ dosage compared to with the lower dosage, although this difference only reached significance occasionally. The TSSc, built by aggregating all symptoms, gradually reduced over time and from the first day of treatment. The two dosage groups started with a very similar severity, and reduced symptoms equally over three days. From day four on, the curves diverged a more effectively reduction of the overall symptomatology with five tablets daily.

#### 3.2.4. Subjective Effectiveness and Acceptance

In the ITT collective, 99% of parents/children subjectively rated the effectiveness of EFJ treatment as “good” or “very good” immediately after resolution of episodes. EFJ treatment was well accepted, as more than 82% of parents would use EFJ tablets again to treat cold symptoms in their children. No significant differences between dosage groups in terms of subjective effectiveness (MH test, *p* = 0.396) and acceptance (FE test, *p* = 0.509) were identified.

#### 3.2.5. Adverse Events and Concomitant Medication

In 130 treatment cycles, 13 children reported 20 mostly mild and self-limiting adverse events (AEs), corresponding to one patient experiencing a single AE per 10 treated episodes. We recorded eight AEs among six children (16.7%) in the low-dose group, and 12 AEs from seven children (21.9%) in the high-dose group (FE test, *p* = 0.759). None of the AEs were serious (SAE), and none showed any causal relationship to the study medication (ADR). A total of 13 out of the 20 AEs were cases of scarlet fever, chicken pox, herpes zoster, urinary tract infections, conjunctivitis, bronchitis, otitis media or tonsillitis. Three out of 20 AE were cases of gastrointestinal complaints such as vomiting, gastrointestinal pain, and celiac disease. Two out of 20 AEs were cases of cold associated complaints including fatigue and flu-like unspecific illness. One out of 20 AEs was due to feelings of dizziness and one out of 20 AEs was due to a disease of the Eustachian tuba.

Concomitant medication used for cold treatment-including analgesics/antipyretics, and prescribed antibiotics was reported for seven children (21.9%) in the high-dose group and eight children (22.2%) in the low-dose group (FE test, *p* = 1.0). Only six cold episodes (4.62%) necessitated antibiotic treatment: Three in the low-dose group and three in the high-dose group (FE test, *p* = 1.0).

## 4. Discussion

Being the population affected most by RTIs and associated complications, children are in great need of effective and safe therapeutics. For this purpose, 79 healthy children were included to treat upcoming cold episodes with either three or five Echinaforce Junior tablets (EFJ) daily for up to 10 days. A total of 130 cold episodes were reported in 68 children overall during the 5.3 months of observation, encompassing a full cold and flu season.

We found that EFJ tablets had an excellent safety profile for the acute treatment of RTIs. Both EFJ dosages elicited exceptionally positive tolerability ratings (“good” or “very good”) from physicians and from parents/children. This matched well with the low occurrence of AEs, of which none was related with the treatment, despite the close-meshed control instances in this trial.

This study tested EFJ under real life conditions comparing dose-dependent effects of two regulatory approved dosages. In this regard, EFJ-treated cold episodes lasted, on average, 7.5 ± 3.5 days. Cold durations reported in literature are subject to variability due to heterogeneity in terms of recruitment setting, time-point of presentation, studied cold symptoms, applied treatment, and endpoint definitions. We have chosen Grüber et al. [25] and Thompson et al. [8] as references because they systematically investigated duration of untreated acute respiratory tract infections and optimally reproduce our study conditions. Grüber et al. stated that untreated cold episodes in children of ≤ 7 years of age last, on average, 13 days [25], while others have reported durations of 8–14 days based on the analysis of multiple clinical trials [8]. Hence, when comparing with data from literature, cold duration upon EFJ treatment appears to be shorter. Likewise, EFJ dose increase from three to five tablets daily further reduced the episode duration by 1.2 days in the intention-to-treat (ITT) and by 1.7 days in the as-treated collective (AT), further strengthening the observed dose-effect correlation (Table 2).

By day 10 of EFJ-treatment, most children in this study had fully recovered from cold symptoms (Figure 3). Again, the rate of non-responders was no more than 8.7% in the high-dose group and 23.5% in the low-dose group (AT collective). In comparison, Thompson et al. reported that approximately 50% of children with untreated cold episodes were still symptomatic by day 10 and reached the 10% non-responder level only after day 15 [8], which is in accordance with observations by Grüber et al. [25]. Parents/children subjectively rated the effectiveness of EFJ predominantly positive (“good” or “very good”) after each episode.

During the study observation (5.3 months), an average of 1.9 cold episodes per patient was recorded. This is approximately 29% fewer cold episodes compared to past seasons (3.1–3.2 colds/year). Since acute colds were treated with EFJ, any preventive benefit was expected to unfold on recurrent infections and relapses only. This observation is in line with recent findings that acute treatment of respiratory infections with *Echinacea* extracts may have a positive effect on the infection recurrence rate [26]. Effects such as immune system modulation, antiviral activity, and anti-inflammatory activity might contribute to the observed clinical benefits in particularly susceptible individuals, here in children [19,27]. For this reason, we analyzed overall occurring episodes including recurrences. EFJ-treated children in our study experienced an average of 13.2 cumulative sick days during the 5.3-month observation time. Roughly extrapolated to a full year, this represents approximately 16 cumulative sick days in a full year considering reported monthly cold incidence rates [25]. This is clearly fewer than the average of 20.1 cumulative sick days per year reported for children of ≤ 7 years old in the absence of interventions [25].

Mainly, children treated with the higher dosage exhibited a milder overall course (Figure 4). For example, Thompson et al. showed that cough symptoms prevailed for several days and maintain 50% of the initial severity at day 10 if untreated [8]. In our present study, the lower EFJ dose yielded effective relief of coughing from day five on, and the higher EFJ dose provided effective relief from day two on.

Primary care physicians in Switzerland apply antibiotics to 20.4% of respiratory tract infections, [28,29], in contrast to an overall prescription rate of 4.62% under the conditions in this study. We speculate that the rather low antibiotic prescription rate observed was due to decreased cold duration, severity of symptoms, and recurrence rates in *Echinacea*-treated children, as has previously been reported for adults with the same treatment [26,30].

### Limitations of the Study

For unknown reasons, we clearly fell short of the target sample size of recruited children; however, the number of recorded cold episodes was sufficient to reach acceptable statistical power. The two dosage groups significantly differed in their age and body variables by co-incidence, but had similar cold susceptibilities (as per history). Hence, the children in both treatment groups were comparable with respect to the studied indication. The authors state that this study does not represent a randomized controlled and double-blinded study, but it displays treatment benefits between two approved dosages of *Echinacea* under real life conditions using a pragmatic approach. Due to the high relevance for individual patients, society, and healthcare systems, we emphasize that the presented findings should be further confirmed by clinical studies that include a distinctly larger pediatric patient collective following a rigorous study design.

## 5. Conclusions

The data presented in this study underpins the advantageous safety profile of Echinaforce Junior tablets and provides evidence for clinical effects to treat common cold symptoms in children. An increase of the daily EFJ dose from three to five tablets seemed to shorten the average cold duration by up to 1.7 days and after 10 days resolved >90% of episodes completely. The overall need for antibiotics under the study conditions was low. Pediatricians may consider EFJ as a valuable and safe option for the acute treatment of respiratory tract infections in their young clients.

## Figures and Tables

**Table 1:** Baseline demographic characteristics of all children included in the safety collective (SAF) and intention-to-treat collective (ITT).

Patient Collective	SAF		ITT	
EFJ ^1^ Dosage Group	3 × 1	5 × 1	3 × 1	5 × 1
N	36	32	32	31
Age in years, mean (±SD)	7.5 (2.62)	5.9 (1.85)	7.4 (2.65)	5.9 (1.88)
Height in cm, mean (±SD)	125.6 (20.2)	115.6 (13.0)	125.2 (20.3)	115.5 (13.2)
Body weight in kg, mean (±SD)	25.9 (10.4)	20.5 (5.2)	25.6 (10.8)	20.5 (5.2)
Past cold episodes ^2^, n, mean (±SD)	3.1 (0.17)	3.2 (0.21)	3.0 (0.19)	3.1 (0.20)
Kindergarten/School (%)	88.9	78.1	87.5	77.4

^1^ EFJ: Echinaforce Junior Tablets. ^2^ Per season.

**Table 2:** Average overall episode duration and cumulative number of sick days per patient in cases where treatment was indicated for the intention-to-treat (ITT) and as-treated (AT) collectives.

		Daily Dosage Regimen		
Collective	Parameter	3 × 1 EFJ ^1^	5 × 1 EFJ ^1^	Wilcoxon Test (*p* Value)
ITT ^2^	Number of cold episodes	61	59	
	CSD ^4^ per study group, days ± SD	494	410	0.022
	CSD ^4^ per patient, days ± SD	13.9 ± 6.41	12.5 ± 6.88	0.362
	Episode duration, days ± SD	8.10 ± 3.52	6.90 ± 3.48	0.046
	Responder/non-responder rate, % ^5^	80.3/19.7	89.3/10.7	0.21
AT ^3^	Number of cold episodes	51	69	
	CSD ^4^ per study group, days ± SD	433	471	0.0022
	CSD ^4^ per patient, days ± SD	16.0 ± 1.58	13.1 ± 1.19	0.048
	Episode duration, days ± SD	8.50 ± 3.68	6.80 ± 3.28	0.02
	Responder/non-responder rate ^5^, in %	76.5/23.5	91.3/8.7	0.005

^1^ EFJ: Echinaforce Junior Tablets, ^2^ ITT: Intention to Treat collective, ^3^ AT: As treated collective^4^ CSD indicates cumulative number of sick days per study group overall and on average per patient during the time of observation. ^5^ Overall ratio of resolved episodes (responder) vs. unresolved episodes (non-responder) after 10 days of treatment.

## Data Availability

The dataset generated and analyzed during the current study is not publicly available because it includes clinical subject data. Thus, this data may only be publicly shared and presented in aggregate anonymous form. However, the dataset is available from the corresponding author upon reasonable request.
